# Post-COVID-19 Condition Prediction in Hospitalised Cancer Patients: A Machine Learning-Based Approach

**DOI:** 10.3390/cancers17040687

**Published:** 2025-02-18

**Authors:** Sara Mahvash Mohammadi, Mikhail Rumyantsev, Elina Abdeeva, Dina Baimukhambetova, Polina Bobkova, Yasmin El-Taravi, Maria Pikuza, Anastasia Trefilova, Aleksandr Zolotarev, Margarita Andreeva, Ekaterina Iakovleva, Nikolay Bulanov, Sergey Avdeev, Ekaterina Pazukhina, Alexey Zaikin, Valentina Kapustina, Victor Fomin, Andrey A. Svistunov, Peter Timashev, Nina Avdeenko, Yulia Ivanova, Lyudmila Fedorova, Elena Kondrikova, Irina Turina, Petr Glybochko, Denis Butnaru, Oleg Blyuss, Daniel Munblit, Sechenov StopCOVID Research Team

**Affiliations:** 1 Centre for Cancer Screening, Prevention and Early Detection, Wolfson Institute of Population Health, Queen Mary University of London, London EC1M 6BQ, UK; 2 Department of Paediatrics and Paediatric Infectious Diseases, Institute of Child’s Health, Sechenov First Moscow State Medical University (Sechenov University), 119991 Moscow, Russia; 3 University of British Columbia, Vancouver, BC V6T 1Z4, Canada; 4 Tareev Clinic of Internal Diseases, Sechenov First Moscow State Medical University (Sechenov University), 119435 Moscow, Russia; 5 Clinic of Pulmonology, Sechenov First Moscow State Medical University (Sechenov University), 119991 Moscow, Russia; 6 Institute for Cognitive Neuroscience, University Higher School of Economics, 101000 Moscow, Russia; 7 Department of Mathematics and Women’s Cancer, University College London, London WC1E 6BT, UK; 8 Department of Internal Medicine No. 1, Institute of Clinical Medicine, Sechenov First Moscow State Medical University (Sechenov University), 119435 Moscow, Russia; 9 Sechenov First Moscow State Medical University (Sechenov University), 119991 Moscow, Russia; 10 Institute for Regenerative Medicine, Sechenov First Moscow State Medical University (Sechenov University), 119435 Moscow, Russia; 11 Division of Care in Long Term Conditions, Florence Nightingale Faculty of Nursing, Midwifery and Palliative Care, King’s College London, London WC2R 2LS, UK; 12 Research and Clinical Center for Neuropsychiatry, 119334 Moscow, Russia

**Keywords:** post-COVID conditions, cancer patients, machine learning classifiers, predictive modelling

## Abstract

Background: The COVID-19 pandemic has led to widespread long-term complications, known as post-COVID conditions (PCC), particularly affecting vulnerable populations such as cancer patients. This study aims to predict the incidence of PCC in hospitalised cancer patients using the data from a longitudinal cohort study conducted in four major university hospitals in Moscow, Russia. Methods: Clinical data have been collected during the acute phase and follow-ups at 6 and 12 months post-discharge. A total of 49 clinical features were evaluated, and machine learning classifiers including logistic regression, random forest, support vector machine (SVM), k-nearest neighbours (KNN), and neural network were applied to predict PCC. Results: Model performance was assessed using the area under the receiver operating characteristic curve (AUC), sensitivity, and specificity. KNN demonstrated the highest predictive performance, with an AUC of 0.80, sensitivity of 0.73, and specificity of 0.69. Severe COVID-19 and pre-existing comorbidities were significant predictors of PCC. Conclusions: Machine learning models, particularly KNN, showed some promise in predicting PCC in cancer patients, offering the potential for early intervention and personalised care. These findings emphasise the importance of long-term monitoring for cancer patients recovering from COVID-19 to mitigate PCC impact.

## Simple Summary

This study investigates the long-term effects of COVID-19 on cancer patients, who are more vulnerable due to weakened immune systems. Many individuals experience lingering symptoms, referred to as PCC, even after recovering from the initial infection. To better understand this condition, the researchers analysed data from hospitalised cancer patients in Moscow, aiming to predict which patients are most at risk of developing PCC. By applying machine learning models, they identified patterns that can help clinicians detect and manage these long-term symptoms. The findings contribute to improving care for cancer patients affected by COVID-19 and offer insights that could aid the broader medical community in understanding the long-term impacts of the virus.

## 1. Introduction

The global COVID-19 pandemic has placed unprecedented strain on healthcare systems worldwide [1]. While most patients recover from the acute phase of coronavirus disease 2019 (COVID-19), a significant subset continues to experience persistent symptoms [2]. This condition is often referred to as long COVID, post-COVID-19 condition (PCC) [3], post-acute sequelae of SARS-CoV-2 infection (PASC) [4], or patients have been labelled COVID long-haulers [5]. The World Health Organisation (WHO) defined PCC as a condition characterised by a variety of “symptoms that persist for at least three months after the initial onset of COVID-19, lasting for two or more months without an alternative diagnosis” [6]. These symptoms are highly variable and commonly include muscle pain, fatigue, respiratory issues such as shortness of breath, cough, chest pain, neurological symptoms, gastrointestinal problems, and skin manifestations [7,8,9]. Patients are still facing challenges in receiving appropriate recognition, support, medical assessment, and care [10,11].

PCC represents a substantial health burden, affecting an estimated 3 to 5 million individuals [12], and up to 70% of patients who were previously hospitalised during their initial infection may face PCC in the future [8,13,14]. It is estimated that 5% to 15% of cancer patients experience severe acute coronavirus disease (COVID-19) infection, with mortality rates reaching as high as 25% [15]. As patients recover from an initial SARS-CoV-2 infection, new concerns have emerged about mortality and long-term health effects after hospital discharge, especially in relation to PCC [16]. It was suggested that cancer patients may be particularly vulnerable to PCC development due to their compromised immune system and reduced physiological reserve [17,18]. Some of comprehensive reviews highlighted the burden of prolonged symptoms and emphasised the need for detailed research to identify the risk factors and clinical characteristics associated with PCC [19]. There is a lack of research examining the long-term effects of SARS-CoV-2 infection on cancer patients.

Cancer patients are shown to be vulnerable against PCC [20]. The major risk factors for developing PCC among cancer patients were having multiple comorbidities [20,21]. Most common PCC manifestations were fatigue, respiratory symptoms, myalgia, sleep disturbance [20] and gastrointestinal symptoms [22]. Cancer patients hospitalised with COVID-19 infection were shown to have lower survival compared to non-cancer hospitalised patients [13]; however, the prevalence of PCC among survivors at different time points in a year after hospital discharge was shown not to differ significantly from the prevalence of PCC of non-cancer patients hospitalised with COVID-19.

There is a need to develop predictive models for PCC development which can guide future management strategies and address the severe effects of this condition on the quality of life for those impacted [13]. Machine learning has shown promise in clinical prediction tasks, particularly when dealing with complex datasets and heterogeneous patient populations. This cohort study aimed to predict the development of PCC 6 months after hospital discharge in cancer patients admitted to hospitals in Moscow during the pandemic by developing and validating a machine learning model based on clinical features. We evaluated multiple machine learning approaches and compared their performance using a standardised follow-up data collection protocol established by the International Severe Acute Respiratory and Emerging Infection Consortium (ISARIC) Global Paediatric COVID-19 Follow-up Working Group.

## 2. Materials and Methods

### 2.1. Study Design, Setting, and Participants

A longitudinal prospective cohort of adults previously hospitalised with COVID-19 to Sechenov University Hospital Network (four major tertiary university hospitals in Moscow, Russia). Inclusion criteria comprised adult patients (18 years of age) with either reverse transcriptase polymerase chain reaction (RT-PCR) confirmed SARS-CoV-2 infection or clinically confirmed infection when laboratory test results were negative, inconclusive, or unavailable. Comprehensive details on the demographic profiles, hospitalisation requirements, and cohort origination are available elsewhere [23,24].

Acute phase data were extracted from electronic medical records (EMR) and the Local Health Information System (HIS) at the host institutions, using the modified and translated ISARIC WHO Clinical Characterisation Protocol (CCP) [25]. The data collected during the acute phase included baseline demographics, comorbidities at the time of the hospital admission, initial symptoms at admission, chest computed tomography (CT) findings, and disease severity.

Data collection and entry were conducted by a team of trained medical students and physician residents with extensive research experience, using telephone interviews and the Research Electronic Data Capture (REDCap) database [23,24,26]. To ensure data integrity and consistency, the process was closely supervised by senior academic researchers.

Patient follow-up was carried out through structured telephone interviews at approximately 6 (±2) and 12 (±2) months after hospital discharge. The data collection process utilised the Tier 1 ISARIC Long-Term Follow-Up Study Case Report Form (CRF), a standardised tool developed by the ISARIC Global COVID-19 Follow-Up Working Group. To ensure linguistic and conceptual accuracy, the CRF underwent independent forward and backward translation into Russian. The follow-up assessments focused on evaluating changes in patients’ physical and mental health status, systematically identifying new symptoms that developed post-discharge, and recording their onset and duration, as previously described [24].

### 2.2. Data Management

Data collection, storage, and management were conducted using REDCap electronic data capture tools (Vanderbilt University, Nashville, TN, USA), hosted at Sechenov University, alongside Microsoft Excel version 16.93.1 (Microsoft Corp, Redmond, WA, USA) [27,28].

### 2.3. Definitions

PCC definition was aligned with the WHO case definition and for the purpose of this study defined as the presence of any symptom that began within three months post-hospital discharge and persisted for a minimum of two months [6]. Symptom duration was calculated from the hospital discharge date, due to the lack of reliable medical records indicating the initial appearance of symptoms.

Patients were classified as severe cases if required non-invasive or invasive ventilation, or ICU care during the acute COVID-19 phase.

### 2.4. Statistical Analysis

In this study, a comprehensive set of 49 features, describing demographics, symptoms at admission, and preexisting comorbidities, was initially considered for inclusion in the prediction model, comprising two continuous variables and 47 categorical variables. The 49 clinical factors used in this study were selected based on prior research identifying key risk factors for both long-term COVID and cancer outcomes [19,23].

To ensure the robustness of the model, an initial statistical analysis was conducted to identify the most relevant features. Descriptive statistics were calculated for all clinical characteristics. Continuous variables were expressed as medians with interquartile ranges (IQR), while categorical variables were summarised using frequencies and percentages. The association between categorical variables was assessed using Fisher’s exact test. For quantitative variables, the Wilcoxon signed-rank test was employed to compare distributions between groups, as this non-parametric test is particularly suited for data that do not follow a normal distribution. Fisher’s exact test and the Wilcoxon signed-rank test were selected for their suitability in analysing categorical and non-normally distributed continuous data, respectively, ensuring a robust initial assessment of variable associations.

Subsequently, only those features with a *p*-value below the threshold of 0.25 were selected for inclusion in the prediction model. The *p*-value threshold of 0.25 was chosen to allow for a more inclusive selection of features in this exploratory phase, ensuring that potentially predictive features were not prematurely excluded [29]. This approach helps avoid the premature exclusion of meaningful predictors while capturing relevant variables that could contribute to the model’s robustness. Statistical analysis was performed using R version 3.5.1.

### 2.5. Model Implementation and Evaluation

Following feature selection, data standardisation was performed to ensure that all variables, particularly the continuous ones, were on a comparable scale. This step is crucial to prevent features with larger numerical ranges from disproportionately influencing the model.

Considering the potential for class imbalance within the dataset, the Synthetic Minority Over-sampling Technique (SMOTE) [30] was applied to the training data. This technique generates synthetic samples to balance the dataset, thereby enhancing the model’s ability to learn from minority class examples without overfitting. However, it is important to note that while SMOTE improves class balance, it does not address all forms of bias that may exist in the data.

The dataset was then divided into training and testing subsets, with a 70:30 split ratio. This split was carefully stratified to maintain the proportion of the outcome classes across both subsets, ensuring that the training process accurately reflects the distribution of the 139 original data. This stratified sampling approach is particularly important in healthcare datasets, where class imbalance can significantly impact the model’s performance and 141 generalizability.

Following the data preprocessing and feature selection, we applied a range of machine learning classifiers to predict PCC in cancer patients. The classifiers selected for this study include logistic regression, random forest, support vector machine (SVM), and k-nearest neighbours (KNN). In addition to traditional machine learning classifiers, we included a neural network model in the form of a multi-layer perceptron (MLP). The MLP was configured with a single hidden layer consisting of 100 neurons, using the ReLU activation function and the Adam optimizer. The model was trained for a maximum of 500 iterations to ensure convergence. These models were chosen due to their proven efficacy in handling structured healthcare data and their ability to capture complex relationships between features. Additionally, various parameters were adjusted using the grid search method, exploring different values to attain optimal performance.

To assess the performance of each classifier, key metrics were calculated, including the area under the receiver operating characteristic curve (AUC), sensitivity, and specificity. These metrics were selected because they provide a balanced view of the model’s performance in both detecting the condition (sensitivity) and correctly identifying those without the condition (specificity). The 95% confidence intervals (CI) for each classifier’s performance metrics were computed using a bootstrap method with 1000 resamples. This approach ensured a reliable assessment of each model’s predictive capabilities, which is crucial for accurately identifying long-term PCC in cancer patients.

Machine analysis was performed using Python version 3.11.5.

## 3. Results

### 3.1. Descriptive Analysis of Population and Their Clinical Features

The 6-month follow-up interviews were conducted between 21 November 2020 and 10 October 2021, when the dominant SARS-CoV-2 genetic linages in Moscow were Wuhan (March 2020–May 2021) and Delta (May 2021–December 2021). In this study, we aimed to evaluate the long-term effects of COVID-19 on cancer patients, especially given the increased vulnerability of this population. The median time between hospital discharge and follow-up interview was 6.99 months (IQR 6.63–7.29 months), which allowed for an adequate window to assess recovery and lingering symptoms in this population. This follow-up period is considered sufficient to capture both short-term and persistent health issues that could affect cancer treatment and overall recovery.

In total, 205 respondents with oncologic conditions participated in the follow-up survey and were included in the analysis, reflecting a diverse sample of cancer patients affected by COVID-19. Of them, 100 respondents were males (48.8%), providing a fairly balanced gender distribution. The median age was 69 years (IQR 61–76 years), indicating an older patient cohort. A total of 203 respondents (99.0%) required ICU admission during hospitalisation, indicating high rates of severe COVID-19 in this group. The most common pre-existing comorbidities were hypertension (145 cases, 70.7%) and chronic cardiac disease (91 cases, 44.4%), which are frequently observed in cancer patients and may contribute to higher severity of COVID-19 cases in this population. The overall prevalence of PCC was 53.17%.

A detailed summary of the demographic and clinical characteristics of both groups is provided in Table 1. Continuous variables are presented as medians with interquartile ranges (IQR), while categorical variables are reported as percentages. Ten features with (*p* < 0.25) were identified, highlighting their potential relevance in predicting PCC in cancer patients. The selected features included age, sex, COVID-19 antibodies, cough, runny nose, shortness of breath, headache, confusion, inability to walk, and muscle aches. These features were subsequently utilised for further analysis and model development.

Further analysis highlighted statistically significant differences between the PCC and non-PCC groups. Patients with PCC were more likely to have detectable COVID-19 antibodies at follow-up (41.7% vs. 57.8%, *p* = 0.025). Respiratory symptoms such as cough were also significantly more prevalent in the PCC group (61.5% vs. 81.7%, *p* = 0.002), as was the presence of a runny nose (2.1% vs. 12.8%, *p* = 0.004).

On the other hand, symptoms like shortness of breath, while more common in non-PCC patients (65.6% vs. 75.2%, *p* = 0.166), did not reach statistical significance. Neurological symptoms such as confusion (3.1% vs. 0%, *p* = 0.101) and physical impairments like inability to walk (2.1% vs. 0%, *p* = 0.218) were reported exclusively in the PCC group, although their sample sizes were too small to establish statistical significance. These findings underscore the multifaceted nature of PCC, particularly in vulnerable populations with cancer.

### 3.2. Predictive Model Performance

Using the ten features most critical for discriminating the data, predictive models were developed for assessing PCC in cancer patients. Five machine learning classifiers were evaluated. Table 2 presents the performance metrics for each classifier, including the AUC with 95% confidence intervals, calculated using bootstrapping with 1000 resamples, as well as sensitivity and specificity.

Among the models, the KNN algorithm demonstrated the highest AUC value of 0.80, with a confidence interval ranging from 0.69 to 0.90, indicating strong discriminatory power. KNN also achieved a sensitivity of 0.73 and a specificity of 0.69, reflecting a balanced ability to identify patients with and without PCC across the selected features.

The SVM model achieved an AUC of 0.73, with a sensitivity of 0.87 and a specificity of 0.63, indicating that it performed well in identifying patients with PCC, though it was less accurate in distinguishing patients without PCC. Other models, such as logistic regression and MLP, demonstrated moderate AUC values of 0.70 and 0.68, respectively, with both sensitivity and specificity lower than those of KNN and SVM. The random forest model showed the lowest performance, with an AUC of 0.61 and a specificity of 0.47, indicating limited predictive capacity for distinguishing between patients with and without PCC.

**Figure 1:**
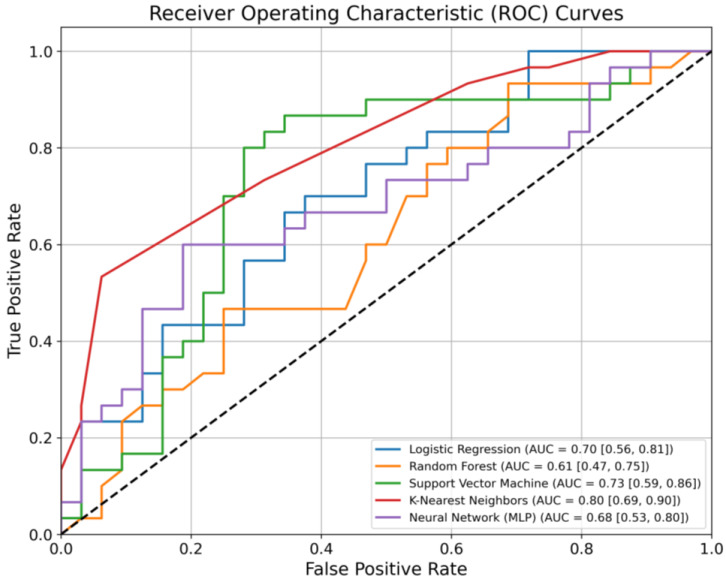
ROC curves comparing the discriminative ability of machine learning models.

Figure 1 visually represents the AUC for each classifier, highlighting their comparative performance. The KNN’s ROC curve, which encloses the largest area under the curve, confirms its superior performance, as indicated by the highest AUC value. All models exhibited some overlap in their confidence intervals, reflecting variability in performance across different resamples. KNN and SVM demonstrated higher sensitivity, whereas the random forest model displayed the largest gap between sensitivity and specificity. These results illustrate notable variability in the predictive performance of the models in identifying PCC in this patient population.

## 4. Discussion

In this study, we aimed to predict the incidence of PCC in cancer patients using a cohort from hospitals in Moscow. Our results highlight significant insights into the long-term consequences of COVID-19 in this vulnerable population, particularly in relation to PCC. Among the models tested, the KNN classifier demonstrated the best performance, with an AUC of 0.80, suggesting its potential as a valuable tool for predicting PCC. This study contributes to the growing body of literature on PCC, addressing the particular vulnerability of cancer patients, who may face increased risk due to compromised immunity and the physiological impact of their underlying condition.

The KNN classifier outperformed other models, with strong discriminatory power (AUC 0.80), while SVM also performed well in terms of sensitivity (0.87), making it particularly effective in detecting patients likely to develop PCC. This suggests that machine learning models, especially KNN, can be effective in identifying PCC risks in cancer patients. The high sensitivity of SVM may be advantageous in clinical settings where under-detection of PCC could lead to prolonged suffering for patients, particularly in a cohort where timely intervention is crucial.

These findings align with previous research highlighting the prevalence of PCC among cancer patients [31,32,33]. The increased risk for PCC in these patients could be due to their weakened immune systems and the systemic inflammation often associated with cancer and its treatment. Additionally, recent studies link immune exhaustion and T-cell dysfunction in long COVID patients, similar to cancer patients. This may impact cancer patients’ long-term recovery from COVID-19 due to a weakened immune response [34].

In this study, the most relevant clinical features selected for model training included pre-existing comorbidities and the severity of the acute COVID-19 infection, which are consistent with previously established risk factors for PCC. While our cohort does not reflect the higher female predominance observed in other studies, this could be due to the specific characteristics of our population, such as the severity of the initial infection and other demographic factors. Future studies with larger, more diverse populations could provide more insight into this aspect.

Our study’s findings are consistent with research emphasising the importance of long-term monitoring for cancer patients recovering from COVID-19 [13,35]. Previous studies have identified a wide range of persistent symptoms in COVID-19 survivors, including fatigue, respiratory issues, and neurocognitive dysfunction. However, few studies have focused specifically on cancer patients, and our study fills this critical gap by leveraging a predictive approach. Notably, the success of KNN and SVM models in predicting PCC resonates with studies using machine learning for COVID-19 outcome prediction in general populations. However, our study is unique in that it applies these techniques to a high-risk, clinically distinct population.

The selection of features such as age, length of stay, and shortness of breath suggests that both demographic factors and the clinical severity of the acute infection are crucial predictors. Age and pre-existing comorbidities are well-known risk factors for severe COVID-19, and this study extends these findings by demonstrating their relevance for predicting PCC in cancer patients. Features such as shortness of breath and cough align with the literature on persistent respiratory symptoms in patients with PCC, especially those with compromised immunity [13,32].

The ability to predict PCC in cancer patients using machine learning offers a promising approach to enhancing post-COVID care. Identifying high-risk patients could allow for early intervention, personalised rehabilitation programmes, and closer monitoring, which may mitigate the long-term burden of PCC. Furthermore, by using widely available clinical data, the models developed in this study could be integrated into existing healthcare systems to streamline patient assessments.

Moreover, KNN’s high predictive power suggests that it could be implemented as a decision-support tool in clinical environments, helping healthcare providers prioritise follow-up care for patients at the highest risk of developing PCC. Given the growing recognition of PCC as a significant healthcare challenge, our study’s findings could influence clinical guidelines, advocating for systematic long-term monitoring of cancer patients with PCC.

This study has several limitations that should be considered when interpreting the results. First, the relatively small sample size and the focus on a specific population (cancer patients in Moscow) may limit the generalisability of the findings to other populations or regions. Additionally, the use of a predefined set of 49 clinical features, based on the prior literature, may have excluded other potentially relevant variables that could enhance model performance. Moreover, some symptoms, such as confusion and inability to walk, had small sample sizes and did not reach statistical significance, which may have affected the conclusions about their relevance. Although our model demonstrated strong predictive performance, its clinical applicability requires validation in prospective cohorts to ensure its effectiveness in practice.

Future research should explore larger, more diverse cohorts and include a broader range of clinical and demographic variables. In particular, increasing sample sizes for less common symptoms could provide more robust insights into their role in predicting PCC. Moreover, our study relied on cross-sectional follow-up data, which limits the ability to capture the temporal progression of symptoms. The integration of longitudinal data could improve the predictive accuracy of models by capturing the temporal evolution of symptoms and their relationship with PCC. Additionally, incorporating biomarker analysis and mechanistic studies could provide deeper insights into the pathophysiology of PCC in cancer patients. Finally, future research could explore the integration of these models into real-time clinical decision-support systems to evaluate their effectiveness in improving patient outcomes.

## 5. Conclusions

In conclusion, this study contributes to the growing body of research aimed at understanding and predicting PCC, particularly among cancer patients. As the long-term impacts of COVID-19 continue to emerge, targeted predictive models like the one developed in this study could play a crucial role in guiding clinical decision-making and improving patient care. The findings underscore the importance of long-term monitoring and personalised care for cancer patients recovering from COVID-19, particularly in preventing and managing the sequelae of PCC.

## Figures and Tables

**Table 1:** Comparison of PCC and non-PCC groups in 6 months after hospitalisation with their corresponding *p*-values.

Variable	PCC	Non-PCC	*p*-Value
Continuous variables			
Length of stay, median [IQR]	15.0 [12.0–18.25]	15.0 [13.0–21.0]	0.299
Age, median [IQR]	67.5 [59.75–73.25]	70.0 [63.0–78.0]	0.096
Categorical variables			
Sex			
Male	57 (59.4%)	43 (39.4%)	0.05
Female	39 (40.6%)	66 (60.6%)	
ICU	95 (99%)	108 (99.1%)	1
Antibodies found	40 (41.7%)	63 (57.8%)	0.025
Symptoms at hospital admission			
Fever	90 (93.8%)	101 (92.7%)	0.789
Cough	59 (61.5%)	89 (81.7%)	0.002
Sore throat	6 (6.2%)	6 (5.5%)	1
Runny nose	2 (2.1%)	14 (12.8%)	0.004
Wheezing	10 (10.4%)	11 (10.1%)	1
Shortness of breath	63 (65.6%)	82 (75.2%)	0.166
Lower chest wall indrawing	0 (0%)	0 (0%)	
Chest pain	8 (8.3%)	12 (11%)	0.639
Conjunctivitis	1 (1%)	1 (0.9%)	1
Lymphadenopathy	2 (2.1%)	2 (1.8%)	1
Headache	17 (17.7%)	28 (25.7%)	0.18
Loss of smell	16 (16.7%)	23 (21.1%)	0.478
Loss of taste	9 (9.4%)	11 (10.1%)	1
Seizures	0 (0%)	0 (0%)	
Fatigue	85 (88.5%)	94 (86.2%)	0.678
Anorexia	15 (15.6%)	18 (16.5%)	1
Altered consciousness/confusion	3 (3.1%)	0 (0%)	0.101
Muscle aches	10 (10.4%)	20 (18.3%)	0.118
Joint pain	4 (4.2%)	5 (4.6%)	1
Inability to walk	2 (2.1%)	0 (0%)	0.218
Abdominal pain	0 (0%)	2 (1.8%)	0.5
Diarrhoea	14 (14.6%)	14 (12.8%)	0.839
Vomiting	7 (7.3%)	14 (12.8%)	0.25
Rash	1 (1%)	1 (0.9%)	1
Bleeding	2 (2.1%)	1 (0.9%)	0.601
Comorbidities			
Chronic cardiac disease	43 (44.8%)	48 (44%)	1
Hypertension	70 (72.9%)	75 (68.8%)	0.542
History of revascularization	8 (8.3%)	12 (11%)	0.639
Chronic pulmonary disease	8 (8.3%)	14 (12.8%)	0.368
Asthma	5 (5.2%)	5 (4.6%)	1
Chronic kidney disease	9 (9.4%)	14 (12.8%)	0.509
Obesity	20 (20.8%)	27 (24.8%)	0.618
Moderate liver disease	3 (3.1%)	3 (2.8%)	1
Mild liver disease	4 (4.2%)	3 (2.8%)	0.708
Asplenia	0 (0%)	0 (0%)	
Chronic neurological disorder	7 (7.3%)	5 (4.6%)	0.553
Chronic hematologic disease	8 (8.3%)	6 (5.5%)	0.581
Diabetes Mellitus	28 (29.2%)	26 (23.9%)	0.429
Rheumatologic disorder	3 (3.1%)	6 (5.5%)	0.506
Dementia	2 (2.1%)	2 (1.8%)	1
Tuberculosis	1 (1%)	0 (0%)	0.468
Malnutrition	2 (2.1%)	2 (1.8%)	1
Smoking	13 (13.5%)	9 (8.3%)	0.262

**Table 2:** Performance metrics of various machine learning models for predicting PCC in cancer patients.

Model	AUC (95% CI)	Sensitivity	Specificity
Logistic Regression	0.70 (0.56–0.81)	0.67	0.66
Random Forest	0.61 (0.47–0.75)	0.7	0.47
Support Vector Machine	0.73 (0.59–0.86)	0.87	0.63
K-nearest Neighbours	0.80 (0.69–0.90)	0.73	0.69
Multi-Layer Perceptron	0.68 (0.53–0.80)	0.67	0.53

## Data Availability

Data available upon reasonable request.

## References

[1] Chen C., Haupert S.R., Zimmermann L., Shi X., Fritsche L.G., Mukherjee B. (2022). Global prevalence of post-coronavirus disease 2019 (COVID-19) condition or long COVID: A meta-analysis and systematic review. J. Infect. Dis..

[2] Iqbal F.M., Lam K., Sounderajah V., Clarke J.M., Ashrafian H., Darzi A. (2021). Characteristics and predictors of acute and chronic post-COVID syndrome: A systematic review and meta-analysis. EClinicalMedicine.

[3] Wise J. (2021). Long COVID: WHO calls on countries to offer patients more rehabilitation. BMJ.

[4] Nalbandian A., Sehgal K., Gupta A., Madhavan M.V., McGroder C., Stevens J.S., Cook J.R., Nordvig A.S., Shalev D., Sehrawat T.S. (2021). Post-acute COVID-19 syndrome. Nat. Med..

[5] Lancet T. (2020). Facing up to long COVID. Lancet.

[6] Soriano J.B., Murthy S., Marshall J.C., Relan P., Diaz J.V. (2022). A clinical case definition of post-COVID-19 condition by a Delphi consensus. Lancet Infect. Dis..

[7] Brodin P. (2021). Immune determinants of COVID-19 disease presentation and severity. Nat. Med..

[8] Carfì A., Bernabei R., Landi F. (2020). Persistent symptoms in patients after acute COVID-19. JAMA.

[9] Huang C., Huang L., Wang Y., Li X., Ren L., Gu X., Kang L., Guo L., Liu M., Zhou X. (2021). RETRACTED: 6-month consequences of COVID-19 in patients discharged from hospital: A cohort study. Lancet.

[10] Kingstone T., Taylor A.K., O’Donnell C.A., Atherton H., Blane D.N., Chew-Graham C.A. (2020). Finding the’right’GP: A qualitative study of the experiences of people with long-COVID. BJGP Open.

[11] Ladds E., Rushforth A., Wieringa S., Taylor S., Rayner C., Husain L., Greenhalgh T. (2020). Persistent symptoms after COVID-19: Qualitative study of 114 “long Covid” patients and draft quality principles for services. BMC Health Serv. Res..

[12] Tenforde M.W., Devine O.J., Reese H.E., Silk B.J., Iuliano A.D., Threlkel R., Vu Q.M., Plumb I.D., Cadwell B.L., Rose C. (2023). Point prevalence estimates of activity-limiting long-term symptoms among United States adults 1 month after reported severe acute respiratory syndrome coronavirus 2 infection, 1 November 2021. J. Infect. Dis..

[13] Fankuchen O., Lau J., Rajan M., Swed B., Martin P., Hidalgo M., Yamshon S., Pinheiro L., Shah M.A. (2023). Long Covid in Cancer: A Matched Cohort Study of 1-year Mortality and Long COVID Prevalence Among Patients With Cancer Who Survived an Initial Severe SARS-CoV-2 Infection. Am. J. Clin. Oncol..

[14] Cabrera Martimbianco A.L., Pacheco R.L., Bagattini Â.M., Riera R. (2021). Frequency, signs and symptoms, and criteria adopted for long COVID-19: A systematic review. Int. J. Clin. Pract..

[15] Fillmore N.R., La J., Szalat R.E., Tuck D.P., Nguyen V., Yildirim C., Do N.V., Brophy M.T., Munshi N.C. (2021). Prevalence and outcome of COVID-19 infection in cancer patients: A national Veterans Affairs study. JNCI J. Natl. Cancer Inst..

[16] Lee L.Y., Cazier J.B., Angelis V., Arnold R., Bisht V., Campton N.A., Chackathayil J., Cheng V.W., Curley H.M., Fittall M.W. (2020). COVID-19 mortality in patients with cancer on chemotherapy or other anticancer treatments: A prospective cohort study. Lancet.

[17] Finn O. (2012). Immuno-oncology: Understanding the function and dysfunction of the immune system in cancer. Ann. Oncol..

[18] Biswas S.K. (2015). Metabolic reprogramming of immune cells in cancer progression. Immunity.

[19] Pazukhina E., Andreeva M., Spiridonova E., Bobkova P., Shikhaleva A., El-Taravi Y., Rumyantsev M., Gamirova A., Bairashevskaia A., Petrova P. (2022). Prevalence and risk factors of post-COVID-19 condition in adults and children at 6 and 12 months after hospital discharge: A prospective, cohort study in Moscow (StopCOVID). BMC Med..

[20] Xu H., Lu T., Liu Y., Yang J., Ren S., Han B., Lai H., Ge L., Liu J. (2025). Prevalence and risk factors for long COVID among cancer patients: A systematic review and meta-analysis. Front. Oncol..

[21] Debie Y., Palte Z., Salman H., Verbruggen L., Vanhoutte G., Chhajlani S., Raats S., Roelant E., Vandamme T., Peeters M. (2024). Long-term effects of the COVID-19 pandemic for patients with cancer. Qual. Life Res.

[22] Dagher H., Chaftari A.M., Subbiah I.M., Malek A.E., Jiang Y., Lamie P., Granwehr B., John T., Yepez E., Borjan J. (2023). Long COVID in cancer patients: Preponderance of symptoms in majority of patients over long time period. Elife.

[23] Munblit D., Bobkova P., Spiridonova E., Shikhaleva A., Gamirova A., Blyuss O., Nekliudov N., Bugaeva P., Andreeva M., DunnGalvin A. (2021). Incidence and risk factors for persistent symptoms in adults previously hospitalized for COVID-19. Clin. Exp. Allergy.

[24] Osmanov I.M., Spiridonova E., Bobkova P., Gamirova A., Shikhaleva A., Andreeva M., Blyuss O., El-Taravi Y., DunnGalvin A., Comberiati P. (2022). Risk factors for post-COVID-19 condition in previously hospitalised children using the ISARIC Global follow-up protocol: A prospective cohort study. Eur. Respir. J..

[25] COVID, WHO Core Case Report Form Acute Respiratory Infection Clinical Characterisation Data Tool. 19 April 2020. https://isaric.org/wp-content/uploads/2021/02/ISARIC-WHO-COVID-19-CORE-CRF_EN.pdf.

[26] Munblit D., Nekliudov N.A., Bugaeva P., Blyuss O., Kislova M., Listovskaya E., Gamirova A., Shikhaleva A., Belyaev V., Timashev P. (2021). Stop COVID cohort: An observational study of 3480 patients admitted to the Sechenov University Hospital Network in Moscow City for suspected coronavirus disease 2019 (COVID-19) infection. Clin. Infect. Dis..

[27] Harris P.A., Taylor R., Thielke R., Payne J., Gonzalez N., Conde J.G. (2009). Research electronic data capture (REDCap)—A metadata-driven methodology and workflow process for providing translational research informatics support. J. Biomed. Inform..

[28] Harris P.A., Taylor R., Minor B.L., Elliott V., Fernandez M., O’Neal L., McLeod L., Delacqua G., Delacqua F., Kirby J. (2019). The REDCap consortium: Building an international community of software platform partners. J. Biomed. Inform..

[29] Hosmer D.W., Lemeshow S., Sturdivant R.X. (2013). Applied Logistic Regression.

[30] Chawla N.V., Bowyer K.W., Hall L.O., Kegelmeyer W.P. (2002). SMOTE: Synthetic minority over-sampling technique. J. Artif. Intell. Res..

[31] Gupta A., Jain V., Singh A. (2022). Stacking ensemble-based intelligent machine learning model for predicting post-COVID-19 complications. N. Gener. Comput..

[32] Shakhovska N., Yakovyna V., Chopyak V. (2022). A new hybrid ensemble machine-learning model for severity risk assessment and post-COVID prediction system. Math. Biosci. Eng.

[33] Reme B.A., Gjesvik J., Magnusson K. (2023). Predictors of the post-COVID condition following mild SARS-CoV-2 infection. Nat. Commun..

[34] Saito S., Shahbaz S., Osman M., Redmond D., Bozorgmehr N., Rosychuk R.J., Lam G., Sligl W., Cohen Tervaert J.W., Elahi S. (2024). Diverse immunological dysregulation, chronic inflammation, and impaired erythropoiesis in long COVID patients with chronic fatigue syndrome. J. Autoimmun..

[35] Monroy-Iglesias M.J., Tremble K., Russell B., Moss C., Dolly S., Sita-Lumsden A., Cortellini A., Pinato D.J., Rigg A., Karagiannis S.N. (2022). Long-term effects of COVID-19 on cancer patients: The experience from Guy’s Cancer Centre. Future Oncol..

